# Fabrication of Suspended PMMA-Graphene Membrane for High Sensitivity LC-MEMS Pressure Sensor

**DOI:** 10.3390/membranes11120996

**Published:** 2021-12-20

**Authors:** Norliana Yusof, Badariah Bais, Jumril Yunas, Norhayati Soin, Burhanuddin Yeop Majlis

**Affiliations:** 1 Faculty of Innovative Design and Technology, Universiti Sultan Zainal Abidin, Kuala Terengganu 21300, Malaysia; 2 Department of Electrical, Electronic and Systems Engineering, Faculty of Engineering and Built Environment, Universiti Kebangsaan Malaysia, Bangi 43600, Malaysia; 3 Institute of Microengineering and Nanoelectronics (IMEN), Universiti Kebangsaan Malaysia, Bangi 43600, Malaysia; jumrilyunas@ukm.edu.my (J.Y.); burhan@ukm.edu.my (B.Y.M.); 4 Department of Electrical Engineering, Faculty of Engineering, University of Malaya, Kuala Lumpur 50603, Malaysia; norhayatisoin@um.edu.my

**Keywords:** (PMMA/Gr) membrane, LC-MEMS pressure sensor, microfabrication

## Abstract

The LC-MEMS pressure sensor is an attractive option for an implantable sensor. It senses pressure wirelessly through an LC resonator, eliminating the requirement for electrical wiring or a battery system. However, the sensitivity of LC-MEMS pressure sensors is still comparatively low, especially in biomedical applications, which require a highly-sensitive sensor to measure low-pressure variations. This study presents the microfabrication of an LC wireless MEMS pressure sensor that utilizes a PMMA-Graphene (PMMA/Gr) membrane supported on a silicon trench as the deformable structure. The (PMMA/Gr) membrane was employed to increase the sensor’s sensitivity due to its very low elastic modulus making it easy to deform under extremely low pressure. The overall size of the fabricated sensor was limited to 8 mm × 8 mm. The experimental results showed that the capacitance value changed from 1.64 pF to 12.32 pF when the applied pressure varied from 0 to 5 psi. This capacitance variation caused the frequency response to change from 28.74 MHz to 78.76 MHz. The sensor sensitivity was recorded with a value of 193.45 kHz/mmHg and a quality factor of 21. This study concludes that the (PMMA/Gr) membrane-based LC-MEMS pressure sensor has been successfully designed and fabricated and shows good potential in biomedical sensor applications.

## 1. Introduction

In the past few years, various polymer materials for highly flexible capacitive membranes have been studied by many researchers [1,2,3,4,5,6,7] to replace typical silicon membranes. This vast interest is because most polymers have low elastic modulus, which produces a large displacement in response to pressure variation and produces higher mechanical sensitivity [8]. Extensive research has recognized the graphene role in bio-integrated soft electronics due to its excellent properties. Recently, the use of graphene as a suspended membrane for MEMS devices is gaining more traction among researchers because of its excellent mechanical characteristics as its own nature’s thinnest membrane [9,10], highly elastic deformation, high tensile strength [11], high conductivity and potentially high biocompatibility [10]. Moreover, graphene has a strong adhesive force to various substrates such as silicon nitride and silicon oxide [12] due to its van der Waals forces, large-scale surface and flatness [13]. These properties make the graphene membrane a promising material for the freestanding membrane, which can be applied as pressure sensors. Generally, graphene is always used with polymers such as PMMA or PDMS as a holder to allow it to remain suspended and subsequently form a freestanding graphene membrane [3,4,5,14,15].

In designing an implantable MEMS sensor, several important critical factors need to be considered to achieve the targeted specifications as a high-performance sensor. Some of the requirements that need to be considered during the design process are the appropriate sensing method, measurement range and precision, frequency response, size, material and telemetry consideration. In biomedical applications, especially for implantable sensors, passive telemetry of inductor–capacitor (LC) MEMS pressure sensors is preferable because this passive wireless sensor does not require batteries to operate, making the system more compact [16]. With the advancement in micromachining technologies, miniaturized LC pressure sensors entrenched in resonant theory became viable and received attention. In LC-based systems, the capacitive membrane, which will bear the applied pressure, plays an important role. When external pressure is applied to the membrane, the elastomeric dielectric layer displays distinctive deformity, prompting a variety in the capacitance. A graphene-based membrane was proposed for the LC-MEMS pressure sensor, considering its characteristics and potential for a capacitive pressure sensor. In the LC-MEMS pressure sensor system, a capacitive membrane needs to be attached with a planar microcoil-shaped inductor to form an LC resonator. When coupling to the external coil, this LC resonator will allow the changes of the sensor’s frequency response due to changes in capacitance or inductance, which can be detected remotely.

Several design specifications must be achieved before designing an LC-MEMS sensor for implantable application, such as the sensor size, safe operating frequency range and high sensitivity to allow detection in a low-pressure variation. One of the challenges in designing implantable sensors is to make them as small as possible so that they can be inserted into the human body without harming the health of the user. In terms of the size, MEMS pressure sensor implants should be less than the organ to be placed. Critically, implant sensors have a size of less than 1 cm^2^ [16,17,18,19,20,21,22]. In their studies, Weaver et al. (2010) [23] and Kim et al. (2014) [24] managed to design sensors with diameter sizes of 10 mm and 8 mm, respectively, for pressure detection applications in the bladder. Meanwhile, Li et al. (2020) developed an LC pressure sensor with the dimensions of 3 mm × 15 mm, specifically designed to monitor intracranial pressure (ICP) [25]. This study set the maximum sensor size at 8 mm based on the previous reports for biomedical implantation applications [16]. The sensor should be able to detect low-pressure changes in the range of 0 to 150 mmHg [26] with a frequency response within the range of 10–100 MHz for biomedical applications. Previous research revealed that the 10–100 MHz operating frequencies are safe for in vivo wireless measurements [27]. The sensitivity of the LC-MEMS pressure sensor from previous studies was in the range between 2 to 162 kHz/mmHg [16,17,18,19,20,21,22,25,28]. However, sensitivity values at higher rates are required for more accurate and precise measurements. In the case of implantable pressure monitoring applications, the design specifications for this MEMS pressure sensor are as shown in Table 1.

This study reported the feasibility of using (PMMA/Gr) membranes to measure capacitance changes in a low-pressure sensing environment. These results suggested that the capacitance changes can be successfully adopted in an integrated planar microcoil design, with the potential development of an LC-MEMS pressure sensor for biomedical implantation applications.

## 2. LC-MEMS Pressure Sensor Working Principle

**Figure 1:**
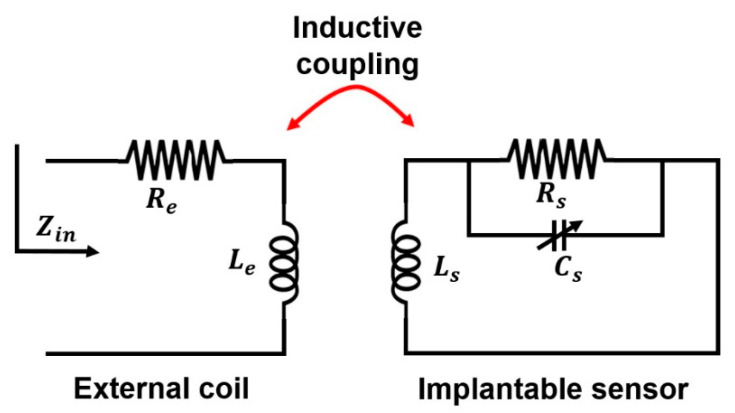
The electrical model of the LC wireless sensing system.

The electrical model of the LC wireless sensing system is shown in Figure 1.

Capacitor, *C_s_*, inductor, *L_s_* and series resistance, *R_s_*, modelled the LC pressure sensor. Inductance, *L_e_*, represents the readout coil, which is in series with resistance, *R_e_*. Faraday’s law states that the resistance of the wireless system, which is similar to the input viewed from the readout coil, can be retrieved from circuit analysis as follows

(1) $$Z_{in}\left(\omega \right)=R_{e}+j\omega L_{e}+\frac{{\left(\omega M\right)}^{2}R_{s}}{{\left(R_{s}-\omega^{2}R_{s}C_{s}L_{s}\right)}^{2}+\omega^{2}L_{s}{}^{2}}\;\;+j\frac{{\left(\omega M\right)}^{2}\left(\omega R_{s}C_{s}\right)\left(R_{s}-\omega^{2}R_{s}C_{s}L_{s}\right)-\omega L_{s}}{{\left(R_{s}-\omega^{2}R_{s}C_{s}L_{s}\right)}^{2}+\omega^{2}L_{s}{}^{2}}$$

where

(2) $$M=k{\left(L_{e}L_{s}\right)}^{1/2}$$

demonstrates the mutual inductance between readout coil and sensor coil, whereas *k* is the inductive coupling coefficient between both coils. The real part of input impedance, with respect to the angular frequency *ω*, is as follows

(3) $$\frac{dR_{e}\left(Z_{in}\left(\omega \right)\right)}{d\omega }=\frac{d}{d\omega }\left\{R_{e}+\frac{{\left(\omega M\right)}^{2}R_{s}}{{\left(R_{s}-\omega^{2}R_{s}C_{s}L_{s}\right)}^{2}+\omega^{2}L_{s}{}^{2}}\right\}=0$$

When solving Equations (1) and (3), the equations relate to the sensor’s resonant frequency [24,25]

(4) $$f=\frac{1}{2\pi }\sqrt{\frac{1}{LC}-\frac{R^{2}}{L^{2}}≅\frac{1}{2\pi \sqrt{LC}}}\;if\;R\ll \sqrt{\frac{L}{C}}$$

In order to obtain the resonance frequency of a pressure-dependent sensor, the real part of the peak impedance from the readout coil was monitored. The readout coil’s wireless sensor system contains an induced peak value at the resonance frequency. Furthermore, the sensor’s resonance frequency was sensitive towards pressure because the sensor comes with pressure-dependent capacitance [29].

As for the value of the quality factor, *Q*, it can be calculated using Equation (5)

(5) $$Q=\frac{\omega_{0}L}{R}$$

where *ω*_0_ is the angular resonance frequency (*ω*_0_ = 2π*f*), *L* is the inductance value and *R* is the coil resistance value.

## 3. Methodology

**Figure 2:**
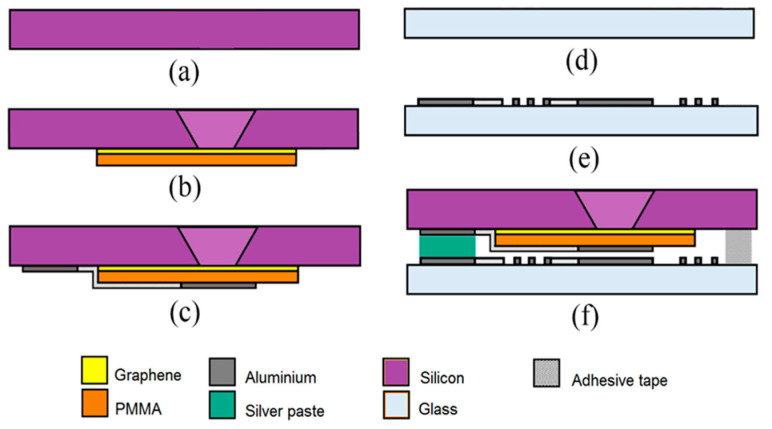
The fabrication process of the LC-MEMS pressure sensor: (**a**) silicon substrate, (**b**) KOH etch and (PMMA/Gr) membrane transfer, (**c**) upper plate aluminium sputter, (**d**) glass substrate, (**e**) microcoil sputter and pattern (**f**) silicon–glass bonding.

Capacitor and inductor were connected to form LC-MEMS pressure sensor, fabricated using silicon bulk micromachining (silicon cavity), CVD graphene transfer process (fabrication of (PMMA/Gr) membrane) and sputtering process (fabrication of microcoil and electrode). The steps involved in the MEMS fabrication process of the LC-MEMS pressure sensor are shown in Figure 2.

First, the suspended (PMMA/Gr) membrane that forms the base of the silicon trench was fabricated, hence, requiring <100> oriented n-type silicon wafer of 4-inch diameter, 525 ± 25 µm thickness, with double-sided 200 nm coated silicon nitride. The photolithography and buffered oxide etchant (BOE) etching process began before fabricating silicon trench using 45 wt% potassium hydroxide (KOH) +10 wt% isopropyl alcohol (IPA) wet etching. The silicon nitride layer was etched to prepare the window pattern, and BOE etching was used to remove the nitride layer using 45 nm min^−1^ at 80 °C etching rate. The sample of this study was immersed in a BOE solution through a double boiling process at 80 °C constant temperature. PMMA (950 PMMA A4, 950 K MW 4 wt%) in anisole by microchemical and a monolayer thin graphene film, provided by University Wafer Inc., were used as the sensor’s membrane.

By employing the wet graphene transfer process, the (PMMA/Gr) layer was then transferred onto the silicon cavity and subsequently onto the etched silicon. Creating a freestanding membrane is vital, and any mishandling during the transfer process may cause the membrane within the cavity area to rupture. Once the suspended membrane (PMMA/Gr) was successfully fabricated, the membrane was then coated with aluminium to make it an excellent electrical conductor as well as provide a current path between the membrane and the connector pad of the MEMS sensor. The direct current (DC) magnetic spark method was used to deposit a layer of aluminium atoms or molecules on the surface of the suspended membrane (PMMA/Gr).

In order to fabricate the microcoil, a Pyrex (7740) glass was used as a substrate to reduce the parallel parasitic capacitance between the microcoil’s turns. After Pyrex glass was cut and cleaned in acetone and methanol, an aluminium layer was then deposited onto Pyrex glass by the DC magnetron spark process. The eight cycles of the deposition process were utilized to deposit the 4 µm aluminium layer, whereby each cycle took 30 min. The sample of the deposited aluminium was patterned with a positive mask using the lithography technique. The sample was then spin-coated with AZ1500 photoresist at 3000 rpm for 30 s and then soft baked for 90 s at 90 °C on the hot plate. The sample was then exposed to UV light for 35 s and soaked in the AZ300K developer for 1 min. Next, the sample was hard-baked at 120 °C for 120 s. In order to etch away the unwanted aluminium pattern area, the sample was dipped in a mixture of 80% phosphoric acid, 10% distilled water, 5% acetic acid and 5% nitric acid.

The samples were soaked in the aluminium etchant for 30 min before dipping it into the acetone solution to remove the photoresist layer. The geometric mask of the coil were: inner size, *di* = 1000 µm; winding number, *n* = 20; trace width, *w* = 50 µm; spacing, *s* = 100 µm. Finally, the silicon and the glass were bonded together to form the LC resonator. The bonding layer was constructed using double-sided adhesive tape A WK6500B (Shenzhen Wenke Electronics Co., Ltd., Shenzhen, China) [30].

## 4. Results and Discussion

### 4.1. Fabrication of Silicon Trench

**Figure 3:**
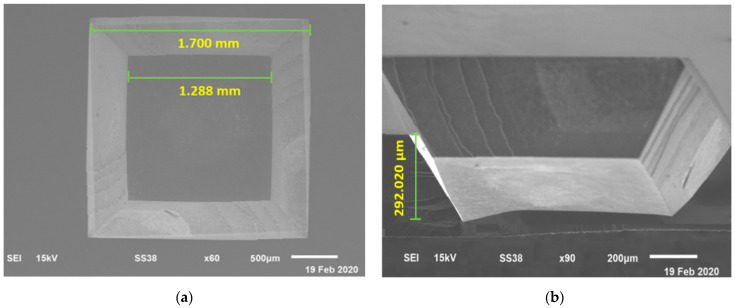
SEM images of: (**a**) Top view of etched silicon; (**b**) Cross-section view of etched silicon.

A wet etch property, including the etching rate and etched silicon thickness, was carefully investigated to ensure a fully etched silicon (hole). This investigation was to determine the required etching time for a fully etched silicon plane (100). Figure 3a,b show the SEM images of the top and cross-section views of the fabricated, etched silicon. The etched surfaces were observed to be smooth and free of hillocks problems. The smooth silicon surface quality was due to the IPA addition to the KOH solution during the wet etching [31]. From Figure 3a, the etched silicon cavity measurement has the dimension of approximately 1288 µm^2^ with an original opening window of approximately 1700 µm^2^ after 4 h of etching. This dimension is in agreement with the equation developed on the tip dimension originated from the surfaces and planes orientation, W = L − (2d/1.414) with W = square-shaped mask, L = dimension of the pyramidal cavity tip and d = thickness of the silicon substrate [32,33]. From Figure 3b, it can be seen that the V-shaped grooves have formed between the (100) and (111) planes. This V-shaped groove was a common effect for KOH wet etching, where the process relied upon the crystallographic directions [34]. After 4 h of etching, the measured silicon thickness was 292 µm. This thickness indicated that the etching rate was about 1.12 µm per minute. Therefore, the additional time for KOH etching required approximately 2.7 h to obtain a fully perforated silicon cavity.

### 4.2. Development and Characterization of Suspended PMMA/Graphene

**Figure 4:**
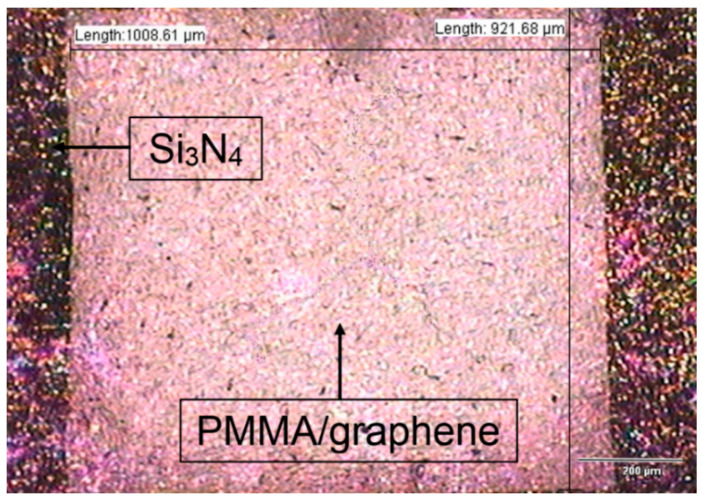
Optical microscopy image of suspended (PMMA/Gr) membrane system.

After the (PMMA/Gr) layer was transferred onto the silicon cavity and coated with an aluminium layer, the suspended membrane was characterized to identify the composition elements of the membrane layer. An optical microscope was used to examine the suspended (PMMA/Gr) membrane, as shown in Figure 4. Monolayer graphene has only 2.3% of light absorption [35], which makes it almost transparent. Observing graphene directly with an optical microscope was quite difficult. However, the top of SiO_2_/Si or Si_3_Ni_4_/Si substrate graphene was noticeably more visible because of slight interferences such as its contrast difference [36,37]. Under the optical microscope observation, the (PMMA/Gr) membrane contained slightly contrasting colours. The darker colour corresponds to the silicon nitride layer, and the lighter colour corresponds to the (PMMA/Gr) membrane. Figure 4 shows that the microscope images of the transferred (PMMA/Gr) were well suspended without any cracks and tears at approximately 1000 µm^2^ silicon cavity area. It was also supported by the strong force of van der Waals.

**Figure 5:**
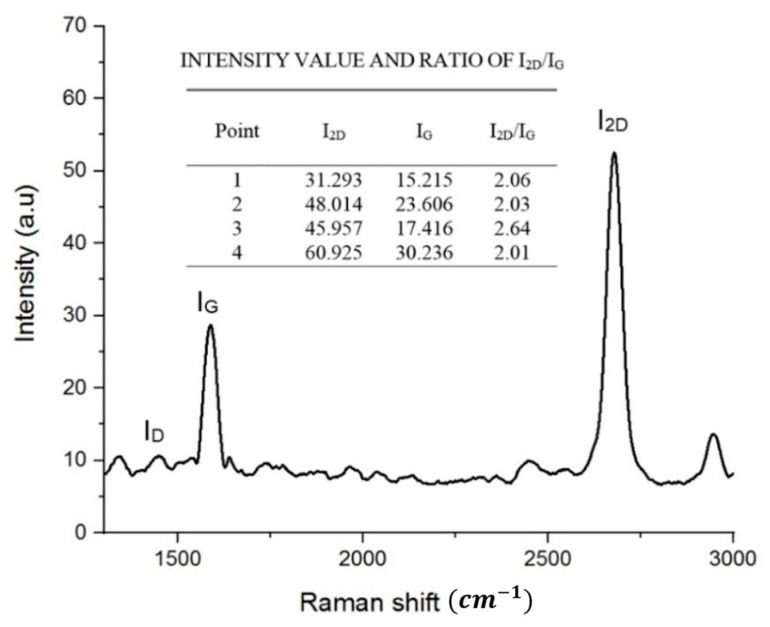
Raman peaks (G and 2D) for (PMMA/Gr) membrane.

The existence of graphene and the number of graphene layers of the (PMMA/Gr) membrane was testified using Raman spectroscopy. The G and 2D bands are at about 1580 cm^−1^ and 2690 cm^−1^, respectively [38,39]. The number of the graphene layer was determined by the peak intensity ratio of 2D to peak G (I2D/IG) with different ratios, i.e., monolayer I2D/IG ~2–3, bilayer 2 > I2D/IG > 1 and multilayer = I2D/IG < 1 [40]. The Raman spectra of freestanding (PMMA/Gr) membrane at four different locations from 1000 cm^−1^ to 3000 cm^−1^ are shown in Figure 5. Additionally, the 2D-band’s graphene intensity was relatively higher than the G-band’s freestanding membrane, indicating that the graphene was significant and could testify to the existence of graphene in the membrane. The inset in Figure 5 also shows the Raman peak intensity values for G and 2D bands for the (PMMA/Gr) membrane. It shows that the I2D/IG ratios in all four different locations were in the range of 2–3, hence, proving that the (PMMA/Gr) membrane has monolayer graphene. Next, the D-peak’s low intensity at 1350 cm^−1^ (ID) concluded that this sample was made of high-quality graphene.

### 4.3. Development and Characterization of Planar Microcoil

**Figure 6:**
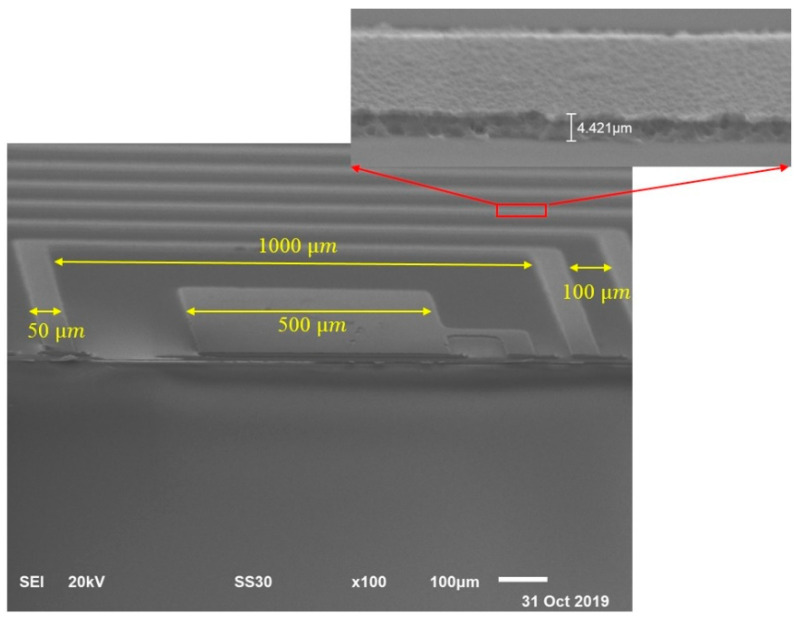
SEM images of fabricated microcoil.

Figure 6 shows the SEM image of the fabricated microcoil. The microcoil was successfully fabricated without any ripple, which can cause openings or shorting of any single trace width. It is crucial to avoid any single trace width being opened or shorted as this will cause the microcoil to be dysfunctional. From Figure 6, the measured dimension of the fabricated microcoil: inner diameter size, di ≈ 1000 µm; trace width w ≈ 50 µm; spacing, s ≈ 100 µm. Additionally, from Figure 6, the thickness of the microcoil was measured to be 4.42 μm. As the total cycle of the sputtering time accumulated to 240 min, the sputtering aluminium rate was deduced to be at 0.02 µm/min. The measurements of the fabricated microcoil were performed using an Agilent 4284A precision LCR meter (Hewlett Packard, Test Equipment Depot, Melrose, MA, USA). The average values of the measured inductance and resistance values were at 2.49 µH and 58.4 Ω for four samples, respectively, at 1 MHz operating frequency. These values resulted in the quality factor, Q, of the microcoil being at 0.27. The 1 MHz operating frequency was chosen due to the maximum operating frequency measured by the Agilent 4284A precision LCR meter. However, the expected value of the Q-factor can be calculated using Equation (4) at higher operating frequency values.

### 4.4. Completed Structure of LC-MEMS Pressure Sensor

**Figure 7:**
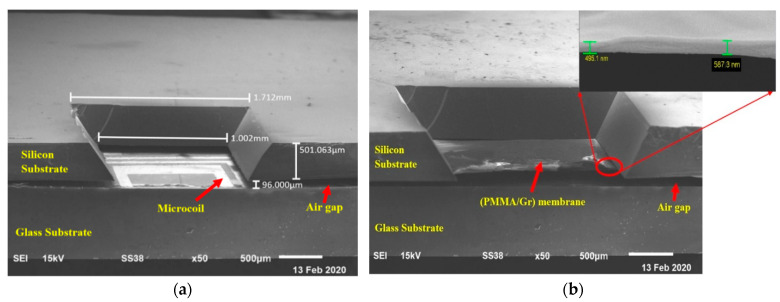
SEM image of LC-MEMS pressure sensor structure: (**a**) Top without the membrane; (**b**) With the suspended (PMMA/G) membrane.

The silicon substrate was then bonded onto a glass substrate, forming an LC-MEMS pressure sensor structure. Figure 7a shows the SEM image of the completed structure of the LC-MEMS pressure sensor structure without the membrane. The membrane part was excluded in Figure 7a to show the existence of the microcoil in the structure. The spacing between the silicon substrate and the glass substrate was adjusted to be approximately 100 µm by a spacer tape and silver paste forming an air gap in the capacitive pressure sensor. The contact pad of the microcoil was bonded to the contact pad of the membrane part with the silver paste to provide an LC tank circuit. As shown in Figure 7a, the measured air gap between the bottom plate (microcoil part) and upper plate (membrane) was 96 µm reaching the target of 100 µm air gap. Figure 7b shows the SEM image of the fabricated LC-MEMS pressure sensor structure with the suspended (PMMA/Gr) membrane. The thickness of the (PMMA/Gr) membrane was measured at approximately 0.5 µm. In Figure 7b, the membrane hid the microcoil part on the glass substrate.

### 4.5. Low-Pressure Testing of LC-MEMS Pressure Sensor

**Figure 8:**
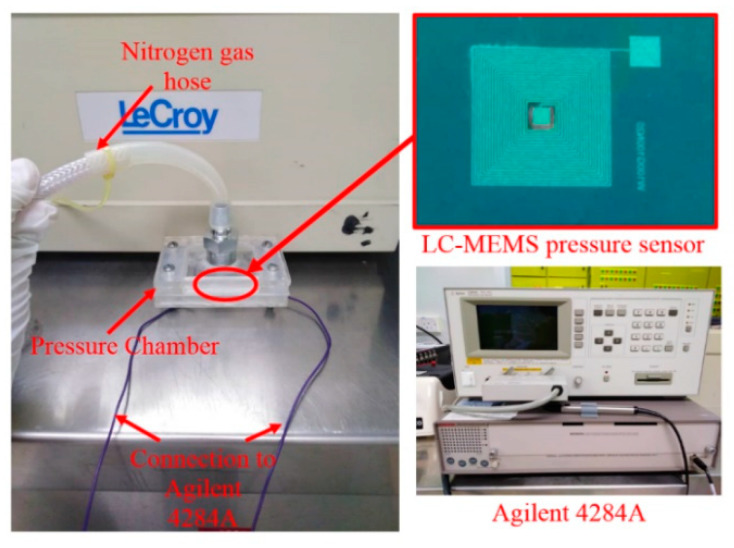
Testing system setup.

**Figure 9:**
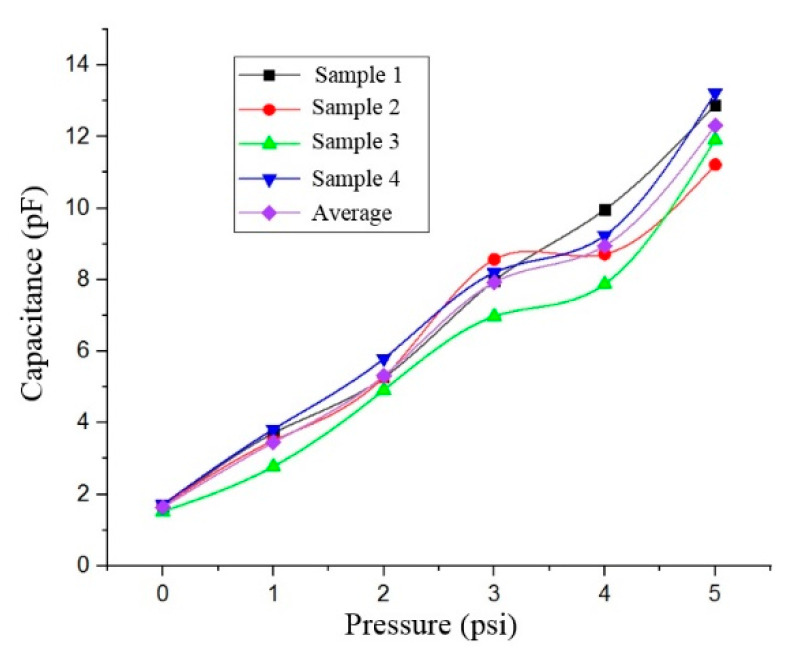
Capacitive responses of the developed pressure sensors.

In order to examine the pressure response of the sensor, low pressure from the nitrogen gas tank was connected to the pressure chamber through a pressure regulator. The experimental setup for the measurement of the LC-MEMS pressure sensor is shown in Figure 8. The gas pressure was controlled using a low range pressure regulator placed between the pressure gauge for the nitrogen gas tank and the pressure chamber. The sensor was placed inside the pressure chamber connected to the Agilent LCR meter to measure the capacitance values as the applied pressure was varied. Figure 9 shows the capacitance plots of four LC-MEMS pressure sensor samples tested individually against the pressure of the flowed gas. From Figure 9, the capacitance changed from 1.64 pF to 12.32 pF for pressure changes within 0 to 5 psi (0~258.57 mmHg). It can also be observed that the change in the capacitance value for sample 1 was more linear than the other three samples. This nonlinearity factor might be due to the noise generated during the pressure testing. It is advisable to use a special chamber for low-pressure sensing to eliminate noise.

**Figure 10:**
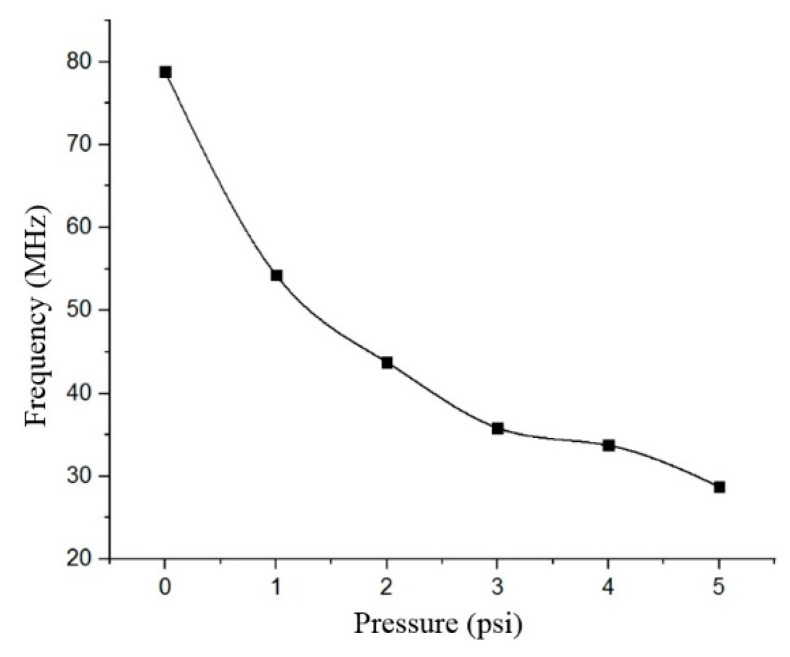
Frequency responses of the developed pressure sensors.

However, it can be observed that the capacitance readings were consistent for all four samples. Referring to the microcoil characterization, the average values for the resistance and inductance of the fabricated microcoil were 58.4 Ω and 2.49 µH, respectively. Since the measured resistance value was very small compared to the ratio of inductance and capacitance (R << L/C), the frequency response to the change in capacitance can be calculated using Equation (3). The change in frequency response to the applied pressure is shown in Figure 10. The value of this frequency change was calculated using Equation (3) by taking the average value of the capacitance from the four sensor samples measured from Figure 9. The inductance value was applied from the electrical characterization results of the microcoil. From Figure 10, the relationship between the frequency response to the varied pressure is fixed within the range of 28.74 to 78.76 MHz. While the expected value of the quality factor at the operating frequency of 78.76 MHz is worth 21 after applying Equation (4). Based on the results obtained, it can be seen that the use of a (PMMA/Gr) material as a membrane in the LC-MEMS pressure sensor structure has successfully increased the sensor’s sensitivity at a high value of 193.45 kHz/mmHg, surpassing the values obtained from previous studies [16,17,18,19,20,21,22,28,29]. Table 2 summarizes the characteristics of the LC-MEMS pressure sensor from the simulation and fabrication results. This sensor has a high-pressure sensitivity as the (PMMA/Gr) contributed to a higher mechanical sensitivity of the sensor [8].

## 5. Conclusions

An LC-MEMS pressure sensor was developed using a MEMS fabrication process, which incorporated a microstructure of suspended (PMMA/Gr) membrane with an aluminium planar micro coil as the wireless-sensing medium. This sensor was designed for pressure monitoring purposes in biomedical implantation applications. Based on the fabrication results, the suspended (PMMA/Gr) membrane was successfully fabricated on the silicon etch cavity. The silicon cavity was realized by the bulk micromachining technique using a 45 wt% KOH + 10 wt% IPA etchant. Meanwhile, the suspended (PMMA/Gr) membrane was realized by employing a graphene wet transfer process using Fe_3_Cl_4_ etchant. The planar microcoil was successfully fabricated by the surface micromachining method utilizing the DC sputtering magnetron method. The fabricated LC-MEMS pressure sensor was tested in a low-pressure environment. The experimental results show that the capacitance varied from 1.64 pF to 12.32 pF as the pressure changed from 0 to 5 psi (0 to 258.57 mmHg). These variations of the capacitance caused the resonance frequency to vary from 28.74–78.76 MHz. From the results and analysis, this LC-MEMS pressure sensor, which applied the PMMA/Gr as a suspended membrane, has produced a high sensitivity value of 193.45 kHz/mmHg suitable for implantable pressure sensing application. It is recommended for future works to focus on the microcoil design, taking into consideration the material selection and process, so that the Q-factor can be improved.

## Figures and Tables

**Table 1:** Design specifications of the LC-MEMS pressure sensor for biomedical implantation applications.

Parameter	Value
Pressure range	0–75 mmHg (normal) ~150 mmHg (abnormal) [26]
Frequency response	10–100 MHz [27]
Size	8 mm × 8 mm [16]
Sensor sensitivity	2–162 kHz/mmHg [16,17,18,19,20,21,22,25,28]

**Table 2:** Summarized characteristics of the fabricated LC-MEMS pressure sensor.

Parameter	Characteristics
Membrane’s material	(PMMA/Gr)
Membrane’s thickness (PMMA/Gr)	0.5 µm
Membrane’s area	1.0 mm × 1.0 mm
Sensor’s size	8 mm
Air gap	96 µm
Capacitance changes	1.64–12.32 pF
Microcoil’s inductance	2.49 µH
Frequency changes	28.74–78.76 MHz
Quality factor	21 (at F = 78.76 MHz)
Sensor’s sensitivity	193.45 kHz/mmHg

## Data Availability

Not applicable.
